# Clinicopathologic Characterization of Diffuse-Large-B-Cell Lymphoma with an Associated Serum Monoclonal IgM Component

**DOI:** 10.1371/journal.pone.0093903

**Published:** 2014-04-04

**Authors:** M. Christina Cox, Arianna Di Napoli, Stefania Scarpino, Gerardo Salerno, Caterina Tatarelli, Caterina Talerico, Mariangela Lombardi, Bruno Monarca, Sergio Amadori, Luigi Ruco

**Affiliations:** 1 Hematology Unit, Sant'Andrea Hospital, Department of Clinical and Molecular Medicine La Sapienza University, Rome, Italy; 2 Pathology Unit, Department of Clinical and Molecular Medicine, Sant'Andrea Hospital, La Sapienza University, Rome, Italy; 3 Clinical Pathology Unit, Department of Clinical and Molecular Medicine Sant'Andrea Hospital, La Sapienza University, Rome, Italy; 4 Hematology Department, Tor Vergata University, Rome, Italy; University of North Carolina at Chapel Hill, United States of America

## Abstract

Recently, diffuse-large-B-cell lymphoma (DLBCL) associated with serum IgM monoclonal component (MC) has been shown to be a very poor prognostic subset although, detailed pathological and molecular data are still lacking. In the present study, the clinicopathological features and survival of IgM-secreting DLBCL were analyzed and compared to non-secreting cases in a series of 151 conventional DLBCL treated with R-CHOP. IgM MC was detected in 19 (12.5%) out of 151 patients at disease onset. In 17 of these cases secretion was likely due to the neoplastic clone, as suggested by the expression of heavy chain IgM protein in the cytoplasm of tumor cells. In IgM-secreting cases immunoblastic features (p<.0001), non-GCB-type (p = .002) stage III-IV(p = .003), ≥2 extra nodal sites (p<.0001), bone-marrow (p = .002), central-nervous-system (CNS) involvement at disease onset or relapse (p<.0001), IPI-score 3–5 (p = .009) and failure to achieve complete remission (p = .005), were significantly more frequent. FISH analyses for BCL2, BCL6 and MYC gene rearrangements detected only two cases harboring BCL2 gene translocation and in one case a concomitant BCL6 gene translocation was also observed. None of the IgM-secreting DLBCL was found to have L265P mutation of MYD88 gene. Thirty-six month event-free (11.8% vs 66.4% p<.0001), progression-free (23.5% vs 75.7%, p<.0001) and overall (47.1% vs 74.8%, p<.0001) survivals were significantly worse in the IgM-secreting group. In multivariate analysis IgM-secreting (p = .005, expB = 0.339, CI = 0.160-0.716) and IPI-score 3–5 (p = .010, expB = 0.274, CI = 0.102–0.737) were the only significant factors for progression-free-survival. Notably, four relapsed patients, who were treated with salvage immmunochemotherapy combined with bortezomib or lenalidomide, achieved lasting remission. Our data suggests that IgM-secreting cases are a distinct subset of DLBCL, originating from activated-B-cells with terminally differentiated features, prevalent extra nodal dissemination and at high risk of CNS involvement.

## Introduction

Diffuse-Large-B-cell Lymphoma (DLBCL) is a biologically heterogeneous entity [1],that is still homogeneously treated with Rituximab-Cyclophosphamide-Adriamycin-Vincristine-Prednisone (R-CHOP) immunochemotherapy [2]. Since the combination of rituximab and CHOP became the gold standard for DLBCL treatment, the International-Prognostic-Index score (IPI-score) has proved to be less powerful [3]. Moreover, the IPI variables [4] do not provide insight into DLBCL biology. A pivotal step in unveiling DLBCL biology and clinical heterogeneity was achieved in 2000 when Alizadeh et al. identified by gene-expression-profiling (GEP) two main groups of DLBCL with substantially different outcomes: Activated-B-cell type (ABC-type) and Germinal-Center-B-cell type (GCB-type) [5]. Since then considerable efforts have been made in order to translate the complexity of GEP-derived information into fewer data readily achievable by routine tests. However, this attempt is still in progress [6], and the choice of shifting towards an upfront intensified treatment remains largely based on the IPI-score or on IPI-derived scores [7], [8]. Notwithstanding, new biomarkers and scores are needed to identify very poor-risk DLBCL sub-groups [9]–[12]. During the course of 2011 we noticed that three newly diagnosed DLBCL patients who shared poor presenting features and early relapse after R-CHOP, had a serum IgM monoclonal component (MC) at disease onset. In the literature only few occasional studies describing IgM-secreting DLBCL associated to haemolytic anemia or other paraproteinemia related events were reported [13]–[15]. In order to find out whether our observation was just an incidental finding, we started to search for similar cases in our database. In 2011 Maurer *et al*. [16], showed that in DLBCL increased serum free light chain (FLC) represented an independent adverse prognostic factor and in 2013 Jardin *et al*. [17] found that an abnormal IgMκ/IgMλ ratio was associated with survival in patients with DLBCL. In this study we have further increased and characterized a series of DLBCL with an associated IgM MC [18], reporting detailed analysis of their clinical, histological and molecular features.

## Methods

### Patients

This is a retrospective study evaluating the incidence of IgM-secreting DLBCL and comparing this subset with a non-secreting control group for clinicopathological features and survival. The study was approved by our Institutional Review Board (procedure code: RS 44/2013, Sant'Andrea Hospital Ethics Committee) and was conducted in accordance with the regulations of health information protection policies. Patients were asked to sign a written consent at disease onset in order to collect their data on an electronic database and to allow further pathological characterization of biological material harvested for diagnostic purposes. Clinical data, including HCV and HBV markers screening, were prospectively collected and obtained from corresponding medical records. A hundred and fifty-one patients (68F/83M, median age 62 years) diagnosed with conventional *de novo* DLBCL [19] between 2005 and February 2013 at Sant'Andrea Hospital of Rome were enrolled in the study. All 151 patients were analyzed for serum protein electrophoresis at disease onset, and those who had a likely monoclonal band in the serum were further investigated by serum immunofixation (methods are fully described in File S1). From the 151 patients a set of 107 consecutive non-secreting DLBCL were selected as control cases for survival analysis. All these cases had a follow up time ≥24 months, unless a DLBCL–related event (i.e. primary refractoriness, relapse or death) had occurred earlier. Immunodeficiency-associated lymphomas, patients who had been previously treated with radiotherapy or chemotherapy for low-grade lymphoma and patients with stage I non-bulky were excluded from the study.

High risk patients younger than 61 years were treated with R-CHOP every 14 days [20], all other patients with R-CHOP every 21 days [2]. Patients with central nervous system (CNS) involvement were treated with R-CHOP every 21 days plus high dose methotrexate at day +8. Patients with IPI score 4–5 or involvement of bone marrow, testis, and craniofacial sites or with involvement of ≥2 extra nodal sites, received intrathecal prophylaxis with 4–6 injections of 12 mg methotrexate.

### HCV and HBV test

Before chemotherapy all patients were tested for hepatitis B surface antigen and its antibody (HBsAg, HBsAb), antibodies to the core antigen (HBcAb, total and IgM), and for hepatitis C virus (HCV) antibodies. Commercially available enzyme immunoassays were used for HBV and HCV determinations (Architect, Abbott Diagnostics, Italy). All cases were tested for hepatitis B virus deoxyribonucleic acid polymerase chain reaction (Amplicor Roche, Italy- lower limit of detection <200 cp/ml). Only cases that were positive for HCV antibodies were further investigated for HCV–RNA (Amplicor Roche, Italy). Either active, inactive or occult HBV carriers [21] were classified as HBV-positive. Cases were considered HCV-positive if HCV antibodies were positive (Table 1).

**Table 1:** Comparison of clinicopathological features in DLBCL subgroups.

Features (cases investigated)	IgM-secreting	Non-secreting	P-value^1^	*IgM+/non secreting*	P-value^2^
	n = 17	n = 134		*n = 34*	
ALC ≤0.840. 10^9^/L^3^ (n = 143)	47%(7/15)	24%(31/128)	.118	23%(7/30)	.265
BULKY>7.5 cm (n = 151)	23%(4/17)	36%(48/134)	.248	41%(14/34)	.320
HBV+ (n = 148)	25%(4/16)	13.6%(18/132)	.261	18%(18/34)	.707
HCV+ (n = 141)	19%(3/16)	13%(16/125)	.368	9%(3/34)	.370
Anemia <12 g/dL (n = 139)	71%(12/17)	42%(51/122)	.036	39%(13/33)	.072
Sex female (n = 151)	70%(13/17)	41%(134)	.008	47%(16/34)	.072
COO^4^: non-GCB-type (n = 41)	100%(17/17)	54.4%(49/90)	.001	85%(29/34)^3^	.357
COO: GCB-type (n = 66)	0%(0/17)	45.5%(41/90)	.001	12%(4/34)	.357
Immunoblastic-Morphology (n = 151)	76%(13/17)	3%(4/134)	<.0001	9%(n = 3/34)	<.0001
Bone Marrow+ (n = 149)	71% (12/17)	28%(37/132)	.002	23.5% (8/34)	.002
CNS^5^ (n = 124)	41%(7/17)	4%(4/107)	<.0001	9%(3/34)	.010
Age >60 (n = 151)	82%(14/17)	54%(75/134)	.040	62%(21/34)	.203
LDH abnormal (n = 148)	56%(9/16)	52%(69/132)	.797	48%(16/33)	.762
Extra nodal sites ≥2 (n = 151)	82%(14/17)	34%(46/134)	<.0001	32% (11/34)	.001
Stage 3–4 (n = 150)	100%(17/17)	68%(91/134)	.003	73%(24/33)	.020
ECOG-PS^6^≥2 (n = 151)	59%(10/17)	33%(44/134)	.057	32%(11/34)	.130
IPI 3–5^7^ (n = 149)	88%(15/17)	55%(73/133)	.009	47%(16/34)	.006
Complete Remission (n = 151)	47%(8/17)	80.5%(108/134)	.005	79%(27/34)	.027

p-value^1^: comparison of clinical features in IgM-secreting and non-secreting DLBCL subgroups.

p-value^2^ comparison of clinical features in IgM-secreting and IgM+/non-secreting DLBCL subgroups.

ALC^3^: absolute lymphocyte count.

COO^4^:: cell of origin based on the Hans algorithm.

CNS^5^ central nervous system involvement at diagnosis or relapse.

ECOG-PS^6^≥2: performance status following the ECOG nomenclature.

IPI 3–5^7^: International prognostic index score 3–5.

### Morphological and Immunohistochemical analyses

Classification and subtyping of all tumors followed the definitions of the 2008 WHO classification of DLBCL [19]. Immunostainings for CD3, CD5, CD20,CD79a, CD10, MUM1, BCL2, BCL6, kappa and lambda light chains and heavy chain IgM (all purchased by DAKO, Denmark) were performed using Dako automated immunostainer (DAKO, Denmark). Immunohistochemistry with anti-MYC (clone Y69, Ventana-Roche, Italy) was conducted using BenchMark Ultra automated immunostainer (Ventana Medical Systems, Tucson, AZ, USA). The Hans algorithm [22] was used in order to classify cases as GCB-type or non GCB-type. Immunostaining results for BCL2 and MYC were recorded as the percentage of positive cells in increments of 10% regardless of the intensity of the staining. Cases were considered as negative if <5% of tumor cells were positive. Immunohistochemical and morphological analyses were independently evaluated by two experienced hematopathologists (LR, ADN). Disagreements were resolved by joint review on a multi-head microscope.

### FISH and molecular analyses

Since the aim of this study was to define the clinicopathologic features of the IgM-secreting subset, the analyses of recurring chromosomal translocations, MYD88 gene mutation, the BCL2/MYC immunhistochemical score, and the presence of EBV-infection were carried out only in the IgM-secreting subset and in a small control group of non-secreting DLBCL cases (Table S1 in File S1). FISH analyses in tissue paraffin sections were carried out with the following probes: MYC dual color break-apart, BCL6 dual color break-apart; IGH/BCL2 dual color fusion (Vysis, Abbott Molecular Inc. US), and BCL2 dual color break-apart (Kreatech Diagnostics, The Netherlands). The cut-off values for the interphase FISH analyses were established following the criteria of Ventura [23]. In situ hybridization for EBV-encoded RNA (EBER) was performed on paraffin sections using Epstein - Barr virus (EBER) PNA Probe/Fluorescein, and FITC/HRP (DAKO, Denmark)

Allele-specific polymerase chain reaction (AS-PCR) was performed using two reverse primers designed to recognize the mutant and the wild-type allele of MYD88 L265P as previously described [24]. The mutant-specific reverse primer was 5′-CCT TGT ACT TGA TGG GGA aCG-3′ and the wild-type-specific reverse primer was 5′-GCC TTG TAC TTG ATG GGG AAC A-3′. The common forward primer was 5′-AAT GTG TGC CAG GGG TAC TTA G-3′. PCR reaction was performed using AmpliTaq Gold PCR Master Mix (Applied Biosystems, Forster City, CA, USA) in a final volume of 25 mL with 50 nM of each primer and 100 ng of DNA. Thermal cycling conditions were as follow: 2 min. at 94°C, followed by 40 cycles of 94°C for 30 s, 57° for 30 s. and a final extension at 68°C for 5 min. The amplified PCR products (159 bp) were separated on 2% agarose gel. One case of Waldenström macroglobulinemia was used as MYD88 L265P mutation positive control (Detailed methodologies are fully described in File S1).

### Statistics

Categorical data were compared using Fisher's exact test and two-sided p-value, whereas for ordinal data, non-parametric tests were used. The definitions of complete response (CR), event free survival (EFS), progression free survival (PFS) and overall survival (OS) were the standard [25]. The actuarial survival analysis was carried out according to the method described by Kaplan and Meier and the curves compared by the log-rank test [26]. The multivariate analyses for survival were carried out by using the stepwise proportional hazards model [27]. Statistical analyses were done with IBM SPSS Statistics 19 (SPSS Inc. Chicago, IL, USA).

## Results

### IgM serum levels at diagnosis and during follow-up

In 19 out of 151 (12.6%) DLBCL a serum monoclonal IgM component was detected. The serum level of monoclonal IgM at diagnosis varied from 2.5 g/dL to 0.22 g/dL (median value 0.42 g/dL). Eleven out of 19 (58%) patients were monitored by serum immunofixation and FLC k/λ ratio during the course of treatment and follow-up. After 1-3 cycles of R-CHOP the monoclonal IgM component disappeared and the FLC k/λ ratio returned to the normal range in all of these patients. Three out of four patients (75%) at tumor recurrence were negative for serum monoclonal IgM and had a normal FLC k/λ ratio. In the remaining case the reappearance of a monoclonal IgM and of FLC k/λ abnormal ratio preceded relapse. Four patients who are in continuous complete remission are persistently negative for both. Two patients with an IgM MC not related to the neoplastic clone (Table 2) showed no disappearance of the IgM MC during and after treatment.

**Table 2:** Clinicopathological features of 19 DLBCL with a serum monoclonal IgM protein.

Cases	Extra nodal sites	Morphology	COO^2^	IgM-I^3^	EBER	LMP1	HIV	MYC-I^4^	BCL2-I^5^	MYC-f^6^	BCL2-f^7^	BCL6-f^8^	MYD88^9^	FUP^-10^	Status
1	CNS^1^, Liver	Immunoblastic	Non -GCB	+	neg	neg	neg	90%	70%	NE^11^	NE	NE	WT^16^	5	Died in progression
2	Marrow, Liver, Lung, CNS	Immunoblastic	Non -GCB	+	neg	neg	neg	50%	40%	NE	NT^12^	NE	WT	5	Died in progression
3	Bone, Marrow, PB, CNS	Immunoblastic	Non -GCB	+	neg	neg	neg	90%	80%	NT	T^13^	T	WT	19	Died in progression
4	Lung, Marrow, CNS	Immunoblastic	Non-GCB	+	neg	neg	neg	70%	neg	NE	NE	NE	WT	1	Early death
5	Bone, CNS, Marrow	Immunoblastic	Non-GCB	+	NE	NE	neg	NE	NE	NT	NT	NT	NE	14	Died in progression
6	Kidney, Gut, Adrenal gland, CNS	Immunoblastic	Non-GCB	+	neg	neg	neg	20%	20%	NT	T	NT	WT	4	Died in progression
7	Intestine, mesenter	DLBCL	Non -GCB	+	neg	neg	neg	40%	>90%	NT	NT	NT	WT	60	CCR1^14^
8	Bone, Marrow, PB, bones	Immunoblastic	Non -GCB	+	neg	neg	neg	40%	50%	NT	NT	NT	WT	18	CCR1
9	Marrow, PB	Immunoblastic	Non -GCB	+	neg	neg	neg	60%	70%	NT	NT	NT	WT	38	CCR1
10	Lung, Marrow	Immunoblastic	Non -GCB	+	neg	neg	neg	10%	60%	NT	NT	NT	WT	29	CCR2^15^
11	Pharynx	Immunoblastic	Non -GCB	+	neg	neg	neg	30%	>90%	NT	NT	NT	WT	41	CCR2
12	Marrow, PB	Immunoblastic	Non -GCB	+	neg	neg	neg	20%	neg	NT	NE	NE	WT	60	CCR2
13	Pharynx, Marrow	Immunoblastic	Non -GCB	+	neg	neg	neg	40%	50–60%	NT	NT	NT	WT	22	CCR2
14	None	T-cell rich	Non -GCB	+	neg	neg	neg	10–20%	80%	NT	NT	NT	WT	35	Died in progression
15	None	Immunoblastic	Non -GCB	+	neg	neg	neg	40%	>90%	NT	NT	NT	WT	1	Early death
16	Kidney, intestine, peritoneum, bone	DLBCL	Non-GCB	+	NE	NE	neg	NE	60%	NE	NE	NE	NE	2	Died refractory
17	Marrow, pharynx, CNS	DLBCL	Non-GCB	+	neg	neg	neg	60–70%	>90%	NT	NT	NT	WT	5	On treatment
18	Marrow, Uterus	Centroblastic	GCB	-	neg	neg	neg	4%	>90%	NT	NT	NE	WT	16	Died in progression
19	Bone	DLBCL	Non-GCB	-	neg	neg	neg	20–30%	60%	NE	NE	NT	WT	7	On treatment

CNS^1^: Central nervous system involvement at diagnosis or during relapse progression.

COO^2^: Cell of Origin defined by the Hans algorithm.

IgM-I^3^: heavy chain IgM expression assessed by immunohistochemistry.

MYC-I^4^: MYC protein expression by immunohistochemistry.

BCL2-I^5^: BCL2 protein expression by immunohistochemistry.

MYC-f^6^: MYC gene translocation by FISH analysis.

BCL2-f^7^: BCL2 gene translocation by FISH analysis, carried out by both IGH/BCL2 and BCL2 break apart probes.

BCL6-f^8^: BCL6 gene translocation by FISH analysis.

MYD88^9^: MYD88 gene analyzed for L265P mutation.

FUP^10^: Follow-up.

NE^11^: Not evaluable.

NT^12^: Not translocated.

T^13^: Translocated.

CCR1^14^:1^st^ Continuous complete remission.

CCR2^15^: 2^nd^ Continuous complete remission.

WT^16^: wild type.

### Pathological Features

**Figure 1:**
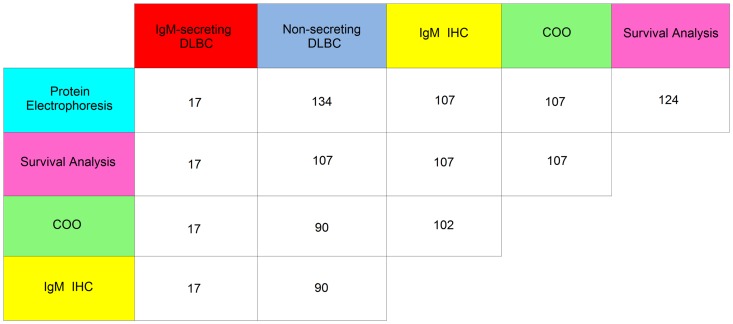
The cross table diagram shows in the colored rectangles the type of analysis that was carried out or the subgroup of DLBCL (non-secreting and IgM-secreting). The white rectangles show the number of cases that match the crossing of horizontal and vertical rows.

One-hundred and seven out of 151 (71%) DLBCL were suitable to be classified for the cell-of-origin (COO) using the Hans algorithm [22]. Of these, 66 out of 107cases (61.6%) were classified as non-GCB-type and 41 out of 107 (38.3%) as GCB-type. The same cases were also analyzed for the expression of cytoplasmic IgM chains, except for five cases (4.7%) in which one of the two assessments was not achievable (Figure 1). Fifty-one out of 107 DLBCL (47.6%) showed IgM expression in the cytoplasm of tumor cells (IgM+ cases); of these, 47 cases (92%) were classified as non-GCB-type and four cases (7.8%) as GCB-type. Within the IgM+ group 17/51 (33.3%) DLBCL had an associated serum IgM monoclonal component. Immunostaining of paraffin tissue sections allowed to detect in the tumor cells the same type of heavy chain IgM, and of light chain κ (n = 15) or λ(n = 2) found in patient's serum. These findings suggest that the serum monoclonal IgM component was related to the DLBCL clone. These 17 cases are from here on referred as IgM-secreting DLBCL (Table 1). The remaining 34/51 cases (66.6%) are referred as IgM+/non-secreting DLBCL. Two additional patients had a monoclonal IgM component in the serum although, tumors stained negative for cytoplasmic IgM (Table 2). All 17 IgM-secreting cases were CD10 negative and MUM1 positive and were classified as non-GCB-type. Moreover, based on the morphology 13 out of the 17 (76%) cases were classified as immunoblastic DLBCL (Figure S1 in File S1) compared to only 4 out of 134 (3%) cases in the control group (p<.0001) and 3 out of 34 (9%) in the IgM+/non-secreting subset (p<.0001) (Table 1). Nine patients out of 134 (6.8%) in the non IgM-secreting group and one out of 17 (5.8%) in the IgM-secreting group had composite lymphoma at diagnosis with the simultaneous presence of DLBCL and a low-grade B-cell lymphoma.

### Molecular analyses

Molecular analyses were performed in the IgM-secreting DLBCL and in a control group of non-secreting DLBCL (Table S1 in File S1). A total of 35 cases were studied for EBV-status, 45 cases for common chromosomal translocations and 30 cases for L265P somatic mutation of MYD88 gene respectively. The results of these analyses did not differ from data reported in the literature [28]–[30]. In the 19 DLBCL patients with a serum IgM MC (Table 2), EBER and LMP1 were evaluable in 17 cases (89.5%) and all of them were negative. None of the 16 evaluable IgM-secreting DLBCL showed MYD88 L265P mutation (Figure S2 File S1). In cases with serum IgM MC FISH analyses were feasible for MYC and BCL2 translocations in 14/19 (74%), and for BCL6 gene rearrangements in 13/19 (68.4%) cases respectively. Two IgM-secreting cases harbored BCL2 translocation; one of these had concomitant translocation of BCL6 gene (Table 2). None of the IgM-secreting cases investigated was found to be rearranged for MYC. Overall, the incidence of chromosomal translocations, and of MYD88 L265P mutation (Figure S2 in File S1) were lower in the IgM-secreting group compared to the control group although the differences were not statistically significant.

### Clinical features and outcome

**Figure 2:**
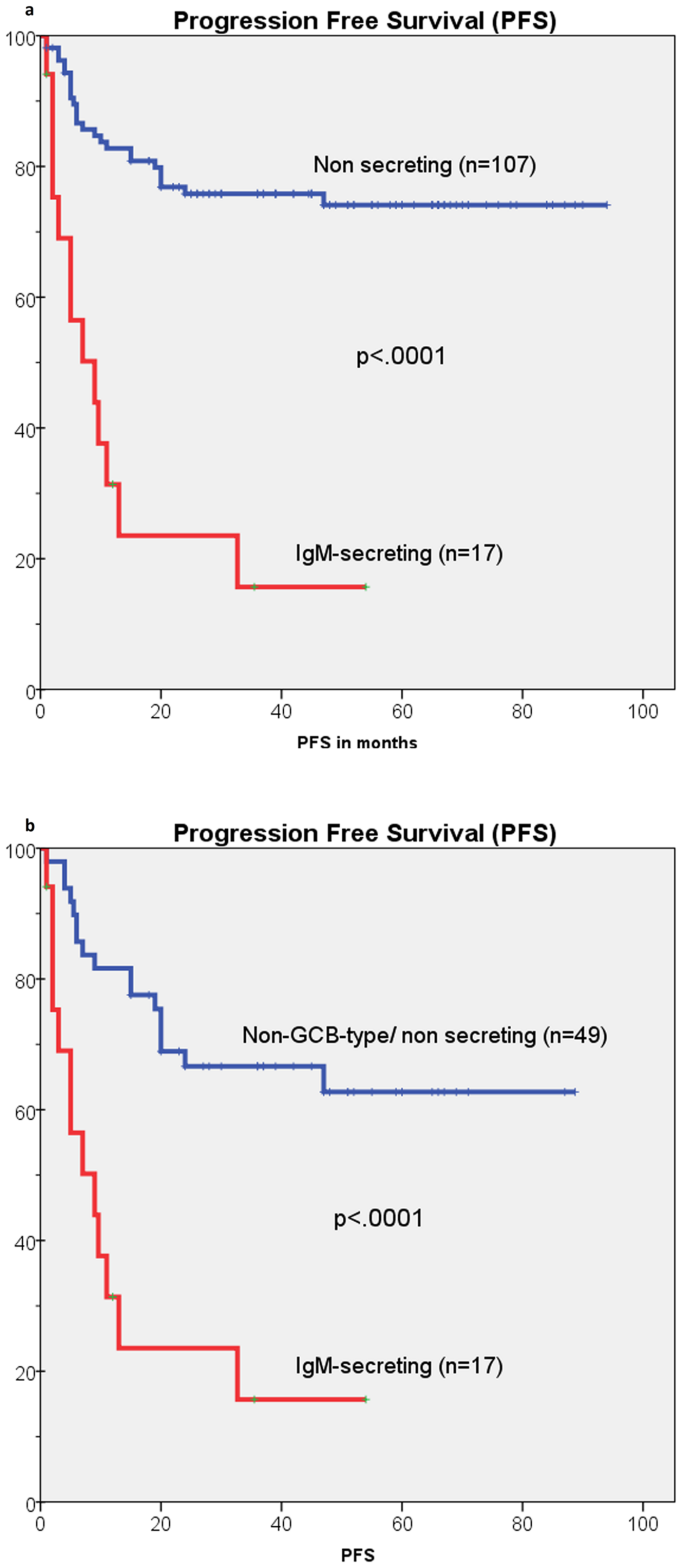
Kaplan-Meier estimates of progression free survival (PFS) in IgM-secreting and in non-secreting DLBCL patients. a) PFS of IgM-secreting and non-secreting DLBCL patients; b) PFS of IgM-secreting and non-GCB-type DLBCL patients.

All the DLBCL (n = 151) were analyzed for clinical characteristics and prognostic scores (Table 1). One-hundred and twenty-four patients out of 151 (82%) with a follow-up ≥24 months or a DLBCL–related event (i.e. primary refractoriness, relapse or death) were considered suitable for survival analysis (Figure 2a). One-hundred and seven patients out of the 124 cases included in the survival analysis (Figure 1) were tested for COO and IgM expression. All the 17 IgM-secreting cases were of the non-GCB-type (100%) in contrast to only 50/90 (55%) in the non-secreting tumors (p = .002).The following clinicopathological features were significantly more frequent in the IgM-secreting group compared to the non-secreting group and also compared to the IgM+/non-secreting subset: immunoblastic morphology, advanced stage of disease, IPI score 3–5, extra nodal involvement ≥2, bone marrow and central nervous system (CNS) involvement, failure to achieve complete remission after R-CHOP. Anemia, female sex and age>60 years were significantly more frequent in the IgM-secreting compared to the non-secreting group (Table 1). Sixteen out of 17 IgM-secreting DLBCL (94%) were *de-novo* DLBCL without a previous history of low-grade lymphoma. One patient was diagnosed with nodal marginal zone lymphoma ten years earlier, but she did not receive any treatment before transformation into DLBCL. One patient had autoimmune hemolytic anemia related to the monoclonal IgM antibody; another with massive kidney infiltration by DLBCL presented a nephrotic syndrome with intact monoclonal IgM in the urine. In the remaining 15 cases no other paraproteinemia-related signs were observed.

Fifteen out of 17 (88.2%) IgM-secreting patients had a DLBCL-related event compared to 36 out of 107 (33.6%) control cases (p<.0001). Nine out of 17 (53%) IgM-secreting patients and 27 out of 107 (25%) control cases died (p<.0001). In the IgM-secreting group, seven patients died with primary refractory or relapsed lymphoma. Two patients died within one month from the start of treatment for toxicity: one patient was in very poor conditions with diffuse meningeal and brain involvement, while the other died of ischemic stroke during treatment. Worthy of note, seven out of 17 IgM-secreting patients (41%) had CNS localization at disease onset (2/17 cases) or during progression/relapse (5/17 cases) (Table 2). One of these patients achieved partial remission on third line treatment with low dose bendamustine and lenalidomide but died in progression after eight months. Eight out of 17 IgM-secreting patients (47%) are alive. Two patients who relapsed within eight months from diagnosis are progression free at +33 and +21 months respectively after second line salvage treatment with bortezomib-rituximab-DHAP [31] followed by high dose therapy (HDT) and peripheral blood stem cell (PBSC) rescue. One patient is progression free at +36 months after second line salvage treatment with rituximab-bendamustine and lenalidomide maintenance [32].One patient who relapsed ten months after diagnosis is in 2^nd^ complete remission at +12 months after rituximab-DHAP and lenalidomide maintenance. One patient, who achieved less than partial remission after four cycles of R-CHOP, is presently in 1^st^ CR after two cycles of RMAD [33] at +15 months. One patient had CNS progression after two cycles of R-CHOP and is responding to high-dose methotrexate. Only two patients are progression free after first line R-CHOP at +60 and +38 months respectively. Of the two patients who had a monoclonal IgM component in the serum but were negative for heavy chain IgM expression by IHC (Table 2), one died with resistant relapse 16 months after diagnosis, while the other is in complete remission seven months after diagnosis (Table 2).

### Survival analysis

**Figure 3:**
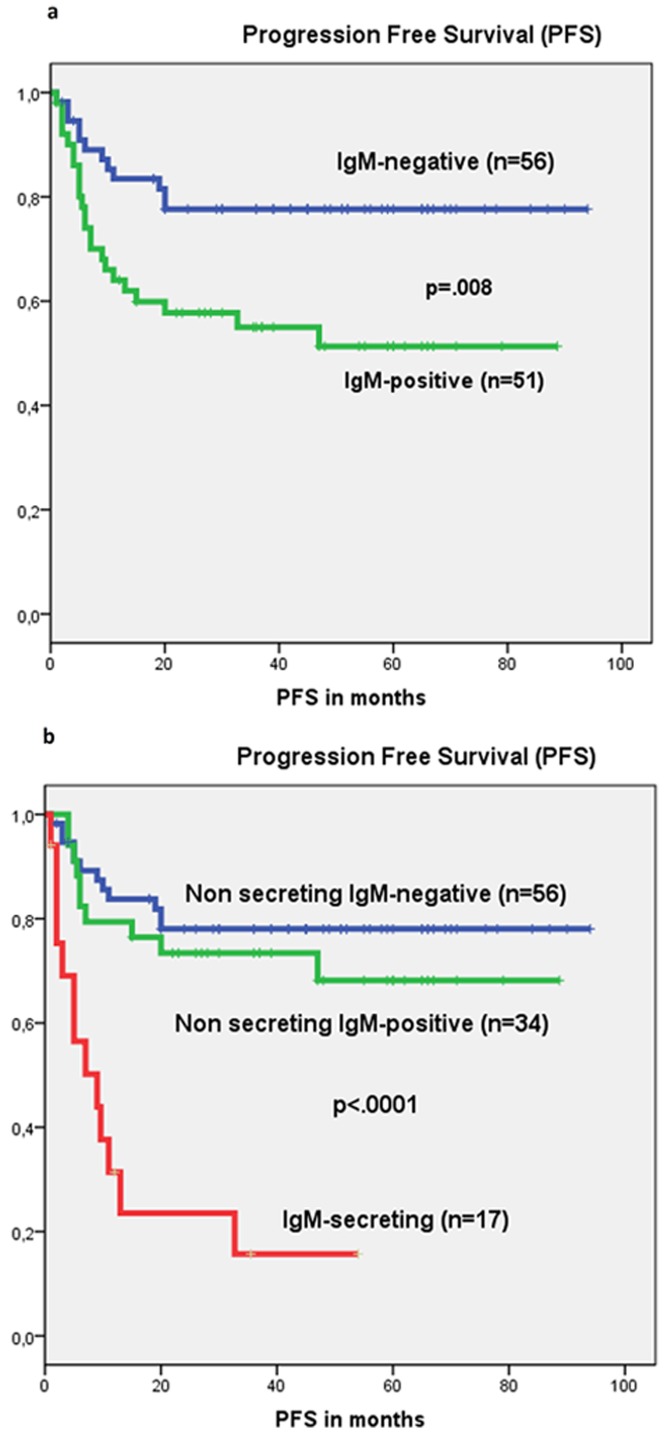
a) Kaplan-Meier estimates of progression free survival (PFS) in patients who are positive for heavy chain IgM (IgM+) by Immunohistochemistry (IHC) and in patients who are negative for heavy chain IgM (IgM-negative) by IHC. b) Kaplan-Meier estimates of PFS in patients negative for heavy chain IgM expression by IHC (IgM-negative), in patients positive for heavy chain IgM expression by IHC but non-secreting (IgM+/non-secreting) and in patients positive for heavy chain IgM expression by IHC and IgM-secreting (IgM-secreting).

The estimated 36-month PFS (23.5% vs 75.7%, p<.0001) (Figure 2a) and OS (47.1% Vs 74.8%, p<.0001) were significantly worse for the IgM-secreting compared to the non-secreting group (Table 3). The differences in survival remained significant even when the IgM-secreting group was compared to the non–GCB-type (Figure 2b). In multivariate analysis (Table 4), IgM-secreting (p = .005, expB = 0.339, CI = 0.160–0.716) and IPI-score 3–5 (p = .010, expB = 0.274, CI = 0.102–0.737) were the only significant factors for PFS; while IPI-score 3–5 was the only significant factor for OS (p = .001, expB = 0.186, CI = 0.071–0.484). A subset of 107/124 (86%) patients who were investigated by IHC for heavy chain IgM expression in tumor samples, were analyzed for the relevance of this factor. The expression of IgM was a significant prognostic factor in univariate analysis (Figure 3a) for PFS (p = .009) and OS (p = .024). However, when patients were subdivided into three sub-groups: 1) IgM-negative (n = 56); 2) IgM+/non-secreting (n = 34) and 3) IgM-secreting (n = 17) survival analysis showed a significant difference only between the IgM-secreting group versus the other two groups (Figure 3b and Figure S3 in File S1).

**Table 3:** Survival analyses comparison for main predictors.

Predictors	36-month rate	P-value
***Progression free survival***		
Non-GCB^1^ (yes vs no)	54.5% vs 85%	<.0001
IgM-secreting (yes vs no)	23.5% vs 75.7%	<.0001
Immunoblastic Morphology (yes vs no)	23.5% vs 75.7%	<.0001
Anemia HB<12 g/dL (yes vs no)	52.7% vs 80.7%	.001
IPI^2^ (0–2 vs 3–5)	52.8% vs 90.4%	<.0001
ALC^3^ ≤0.840. 10^9^/L (yes vs no)	51.6% vs 75.3%	.003
Bone marrow involvement (yes vs no)	46.3% vs 79%	.001
***Overall survival***		
IgM-secreting (yes vs no)	47.1% vs 74.8%	<.0001
Immunoblastic Morphology (yes vs no)	47.1% vs 74.8%	<.0001
IPI (0–2 vs 3–5)	56.9% vs 90.4%	<.0001
ALC ≤0.840. 10^9^/L (yes vs no)	61.3% vs 76.5%	.022
Bone marrow involvement (yes vs no)	58.5% vs 76.5%	.024

Non-GCB^1^ =  Non Germinal Center type, evaluated on the basis of the Hans' algorithm.

IPI^2^ =  international prognostic index scores 0–2 and 3–5.

ALC^3^ =  absolute lymphocyte count.

**Table 4:** Multivariate analyses for the response and survival.

Progression free survival	Exp(B) (95%CI)	P-value
IPI (3–5)^1^	0.274(0.102–0.737)	.010
IgM-secreting	0.339(0.160–0.716)	.005
**Overall survival**		
IPI (3-5)	0.186 (0.071–0.484)	0.001

IPI^1^ (3–5): International prognostic score index value 3–5.

## Discussion

The identification of poor-prognostic subgroups correlated to defined biological tumor characteristics is the aim of modern oncological research. In the case of DLBCL this issue is presently a matter of intense development and debate. Several prognostic factors and scores have been proposed to better stratify patients who would benefit from more intensive treatment than R-CHOP [5], [10], [11]. We had previously reported on a subset of conventional DLBCL associated with a serum monoclonal IgM component characterized by advanced disease and poor prognosis after R-CHOP [18]. In this study we described its clinical, pathological and, molecular features more in depth.

DLBCL with serum IgM MC represented a sizable subset of our series. The majority of these cases were defined as IgM-secreting DLBCL since same type of heavy chain IgM and κ or λ light chains were detected in tumor cells. Most patients had advanced disease, involvement of several extra nodal sites including bone marrow and high IPI-score. Worthy of note, the incidence of CNS involvement at diagnosis or during relapse/progression was surprisingly high. Most of these poor prognostic features remained significantly more frequent in the IgM-secreting subset even when compared to the IgM+/non-secreting subset. Monitoring patients by immunofixation and FLC κ/λ ratio during and after therapy was of little value for predicting relapse. All IgM-secreting cases were classified as non-GCB-type and the great majority showed Immunoblastic morphology.

L265P mutation of MYD88 gene has been reported within 6.5% and 17% of unselected DLBCL [28]–[30]. More recently, this mutation has been shown to be very common in DLBCL originating in extra nodal sites [30]. Since L265P mutation of MYD88 gene was prevalent in IgM-secreting Waldenström macroglobulinemia [24], and it was reported in up to 29% of DLBCL with an ABC-type [34], we expected to find this mutation in the IgM-secreting DLBCL subset. Surprisingly, none of IgM-secreting DLBCL showed L265P mutation of MYD88 gene. This result suggests that other molecular pathways may be involved in IgM-secreting DLBCL [35].

Recurring chromosomal translocations have been reported in DLBCL [36]–[40]. In our series of IgM-secreting DLBCL these were not a distinct feature. None of the tumors showed MYC rearrangement and only two cases harbored BCL2 translocation.

In 2011 Maurer et al [16], showed that increased serum FLC, present in 32% of DLBCL was an independent adverse prognostic factor. More recently, after the introduction of a new sensitive method for immunoglobulin heavy chain detection, a prospective study showed elevated IgMκ or IgMλ or an abnormal IgMκ/IgMλ ratio to occur in 9.3% and 19.1% of DLBCL respectively [17]. Similarly to us, they found a lower PFS and OS in patients with IgM serological abnormalities. Although the two methods are not perfectly comparable, patients with a serum monoclonal IgM detected by immunofixation, as we did in our study, should reasonably have an abnormal IgMκ/IgMλ ratio and possibly also an elevated IgMκ or IgMλ immunoglobulin. The lower sensitivity of our method and the fact that we carried out serum immunofixation only in those patients who had an abnormal protein electrophoresis could explain why the proportion of IgM-secreting DLBCL in our series was somewhat lower compared to that with an abnormal IgMκ/IgMλ ratio described by Jardin et al. [17]. Conversely, Jardin et al. using a very sensitive detection method (Binding Site's Hevylite assay, San Diego, CA, USA) could have possibly found cases with an abnormal IgMκ/IgMλ ratio or an elevated IgMκ or IgMλ without a real clinical significance.

The expression of the heavy chain IgM gene was shown in the Wright signature to be one of the most discriminating genes between GCB and ABC DLBCL subtypes [41]–[42]. It has been also reported that IgM isotype expression in tumor tissues is a more powerful prognostic marker than the Hans algorithm [43]. In our series the expression of heavy chain IgM in tumor cells was found in less than half of the patients. IgM+ cases were mostly classified as non-GCB-type and a third of the cases belonging to this group were also IgM-secreting. Survival analyses of IgM+/non-secreting subset was similar to that of IgM-negative patients. Although we could not analyze all the series by IHC for heavy chain IgM, our data suggests that the poor prognosis attributed to IgM+ cases could be at least in part related to those patients who are IgM-secreting. Notably, monitoring patients by immunofixation and FLC κ/λ ratio during and after therapy was of little value for predicting tumor relapse. Indeed, even in overt relapse, three out of four patients were found negative for serum IgM monoclonal component. It can be speculated that this finding is the result of a clonal evolution of lymphoma cells during progression leading to loss of secretion capability.

Recently, it has been identified a plasmablastic subtype of DLBCL thought to derive from terminally differentiated B-cells [44]. This subset is frequently associated with HIV and EBV infection and is considered a very poor prognostic group. The IgM-secreting subset we characterized, was HIV and EBV negative [45]–[46] and did not show the morphological and immunophenotypic findings of plasmablastic lymphoma. Conversely, the majority of IgM-secreting cases showed immunoblastic features. This finding is in accordance with the notion that secreting capability is acquired by B-cells during end-stage differentiation and that terminally differentiated high grade lymphoma has a poor prognosis [44], [47]–[48].

In our series of IgM-secreting DLBCL, we found a strikingly high incidence of CNS involvement. This finding is in keeping with previous studies showing that primary lymphoma of the central nervous system expresses an IgM isotype [49]–[51], and with the detection of FLC in the cerebrospinal fluid of patients with CNS lymphoma [52]. If our observation will be validated by further studies, an intensified CNS prophylaxis should be recommended in patients with IgM-secreting DLBCL.

Outcome after R-CHOP was disappointing in IgM-secreting patients with most relapses occurring within one year from diagnosis. Interestingly, four patients who relapsed without CNS involvement achieved lasting complete remission when treated with immunochemotherapy combined with biological drugs such as bortezomib or lenalidomide [31]–[32].This fact might suggest that these combinations could be more effective and less toxic than high-intensity treatments in this very poor risk group.

Finally, we speculate that IgM-secretion in DLBCL has since been underrated because it is easily missed by clinicians given the rarity of associated clinical signs, its low entity at diagnosis and rapid disappearance during treatment.

The main limitations of our study pertain to its retrospective nature. IgM expression analysis and COO subtyping were not assessable in all the cases because of the lack of suitable material. Moreover, we classified tumors basing on the Hans' algorithm, which is less sensitive and specific than GEP analysis [5]. We also regret that during follow-up serum immunofixation and FLC k/λ ratio were done only in roughly half of the patients.

In DLBCL patients the identification of reliable prognostic markers that may guide in selecting a more specific treatment is of considerable importance [53]. Widely used and inexpensive routine analysis could easily detect serum monoclonal IgM component. Further confirmation by immunohistochemistry or by other molecular detection assays of IgM expression by tumor cells [43], [54] would allow to identify IgM-secreting DLBCL. We believe that IgM-secreting capability represents a very robust prognostic marker, since it is related to a defined biological characteristic of DLBCL derived from terminally differentiated B-lymphocytes.

## Supporting Information

File S1 Supplementary Materials.
(DOCX)
