# Supplementary material for: Clinicopathologic Characterization of Diffuse-Large-B-Cell Lymphoma with an Associated Serum Monoclonal IgM Component
Source: PLoS One. 2014 Apr 4;9(4):e93903. doi: 10.1371/journal.pone.0093903 (PMC3976325; doi:10.1371/journal.pone.0093903)
Supplement: File S1 — Supplementary Materials. (DOCX) [file pone.0093903.s001.docx]

METHODS

This is a retrospective study evaluating the incidence of IgM-secreting DLBCL and comparing this subset with non-secreting control group and with IgM+/non-secreting subset for clinicopathological features and survival. The study was approved by our Institutional-Review-Board and was conducted in accordance with the regulations of health information protection policies. Patients were asked to sign a written consent at disease onset in order to collect their data on an electronic database and to allow further pathological characterization of biological material harvested for diagnostic purposes. Clinical data, including HCV and HBV markers screening, were prospectively collected and obtained from corresponding medical records. Nineteen patients with a serum monoclonal IgM component were identified within a consecutive series of 151 patients with conventional DLBCL (WHO 2008) diagnosed between January 2005 and February 2013. They were analysed for serum protein electrophoresis at disease onset and those who had a likely monoclonal band in the serum were further investigated by serum immunofixation. Patients were treated with R-CHOP every 14 or 21 days. Patients with IPI score 4-5 or involvement of bone marrow, testis , craniofacial sites or with ≥2 extra nodal site involvement, received intrathecal prophylaxis with 4-6 injections of 12mg methotrexate. Patients with central nervous system (CNS) involvement were treated with R-CHOP-21 plus high dose methotrexate at day +8. All patients were HIV negative. A set of 107 consecutive non-secreting patients were selected from the same clinical series as control cases for survival analysis. All control cases had a follow up time ≥24 months, unless a DLBCL–related event (i.e. primary refractoriness, relapse or death) had occurred earlier, cases who had been previously treated with radiotherapy or chemotherapy for low-grade lymphoma and patients with stage I non-bulky were excluded from the study.

**Morphology, Immunohistochemistry and FISH:**

Morphological analysis was carried out on tissue sections stained with hematoxylin and eosin. Sub typing followed the definitions of the 2008 WHO classification. Paraffin sections were immunostained for CD3 (clone F7.2.38., 1:50, Dako), CD5 (clone SP19, 1:50 Dako), CD20 (clone L26, 1:50, Dako), CD10 (clone 56C6, 1:20, Dako), CD30 (clone Ber-H2, 1:50. Dako), CD79a (clone JCC117, 1:25, Dako), MUM1 (clone MUM1p, 1:25, Dako), BCL2 (clone 124, 1:50, Dako), BCL6 (clone P6-B6p, 1:25, Dako), LMP-1(Dako) kappa and lambda light chains and IgM (Rabbit Polyclonal, 1:20, Dako), using the Dako automated immunostainer (DAKO, Denmark). MYC (clone Y69, Ventana-Roche) immunohistochemistry was performed using the BenchMark Ultra automated immunostainer (Ventana Medical Systems, Tuscon, AZ). All immunostainings and morphological analyses were independently evaluated by two experienced hematopathologists (LR, ADN). Disagreements were resolved by joint review on a multihead microscope. The Hans algorithm (Hans et al, 2004) was used in order to classify cases as GCB-type or non GCB-type. According to the classification proposed by Hans, each case was assigned as GCB or non-GCB based on the expression of CD10, BCL6 and MUM1/IRF4 by tumour cells. The expression was considered positive if at least 30% of the tumour cells stained with the antibody. Immunostaining results for BCL2 and MYC were recorded as the percentage of positive cells in increments of 10% regardless of the intensity of the staining. Cases were considered as negative if <5% of tumor cells were positive. For IgM lymphomas were considered as positive if more than 30% of tumor cells showed a cytoplasmic staining. Cases with values below this threshold were considered weakly positive (+/-) if more than 5% of tumor cells were positive. FISH analysis in tissue sections was carried out with the following probes: dual colour break-apart for MYC and BCL6 genes abnormalities and the dual colour IGH/BCL2 (Vysis, Abbott Molecular Inc. US) and BCL2 break probe (Kreatech Diagnostics, The Netherlands). The cut-off values for the interphase FISH analyses were established following the criteria of Ventura^23^

In situ hybridization for EBV-encoded RNA (EBER) was performed on paraffin sections
using Epstein–Barr Virus (EBER) PNA Probe/Fluorescein and
FITC/HRP (Dako).

Serum immunofixation and protein electrophoresis were performed by capillary zone electrophoresis using equipment (Capillarys protein, Sebia, Italy) and reagents purchased from Sebia. Serum immunofixation and protein electrophoresis were performed using equipment and reagents purchased from Sebia (HYDRASYS, Sebia, Italy). This assay was performed following manufacturer’s instructions. Depending on the type of monoclonal protein, the lower limit of detection for this assay ranges from 0.12 to 0.025 g/dL. Measurement of κ and λ FLC was performed by rate turbidimetry using reagents (FREELITE) purchased by the Binding Site (San Diego, CA), following manufacturer’s instructions. The lower limits of detection for the κ and λ FLC assays are within 0.003 and 0.004 g/dL, respectively.

**AS-PCR Assay for MYD88 L265P mutation**

Allele-specific polymerase chain reaction was performed using two reverse primers designed to recognize the mutant and the wild-type allele of MYD88 L265P as previously described [22]. The mutant-specific reverse primer was 5’-CCT TGT ACT TGA TGG GGA aCG-3’ and the wild-type-specific reverse primer was 5’-GCC TTG TAC TTG ATG GGG AaC A-3’. The common forward primer was 5’-AAT GTG TGC CAG GGG TAC TTA G-3’. PCR reaction was performed using AmpliTaq Gold PCR Master Mix (Applied Biosystems, Forster City, CA, USA) in a final volume of 25 mL with 50 nM of each primer and 100 ng of DNA. Thermal cycling conditions were as follow: 2 min. at 94°C, followed by 40 cycles of 94°C for 30 s., 57°C for 30 s., and 68°C for 30 s., and a final extension at 68°C for 5 min. The amplified PCR products (159 bp) were separated on 2% agarose gel. One case of Waldenström macroglobulinemia was used as MYD88 L265P mutation positive control.

**STATISTICS**

The difference of the two groups was tested with non-parametric methods. Because of the descriptive nature of these comparisons, the p-values were not adjusted for multiple comparisons and the significance level was set at p<0.05. Survival analyses were done by the Kaplan-Meier method. Event-free survival (EFS) was defined as the time from the first treatment to disease progression, start of salvage treatment, additional (unplanned) treatments, relapse, or death from any cause. Progression free survival (PFS) was calculated as EFS, excluding late deaths not related to lymphoma or its treatment. Overall survival (OS) was defined as the time from first treatment to death of any cause. The analyses of factor predicting survival were carried out by the log-rank test. Cox's regression was used for multivariate analyses. The prognostic factors considered were: IPI, IPI dichotomized into two classes (0-2 scores and 3-5 scores), the IPI factors (i.e., age >60, LDH >normal, ECOG >1, stage III/IV, more than two extra nodal sites), bone marrow-involvement; absolute lymphocyte count (ALC) < 0.840x 10^9^/L (Cox et al, 2008) Bulky disease >7.5cm, cell of origin based on the Hans’ algorithm (Hans et al, 2004), the presence of heavy chain IgM by IHC, BCL2 protein expression and double-hit cases basing on IHC results. Relative risks with 95% confidence intervals (CI) and p-values are presented. As for many of the IHC and translocation markers we observed a variable number of cases failing to yield reliable staining/FISH results, the number of evaluable cases varied from one marker to another (Figure 1). Cohens kappa statistic was used to assess the concordance between two different observers (ADN, LR). The IBM SPSS19 package (SPSS Inc. Chicago, IL, USA) was used for all statistical elaborations.

**Figure S1: Morphology and Immunohistochemistry in IgM-secreting DLBCL**
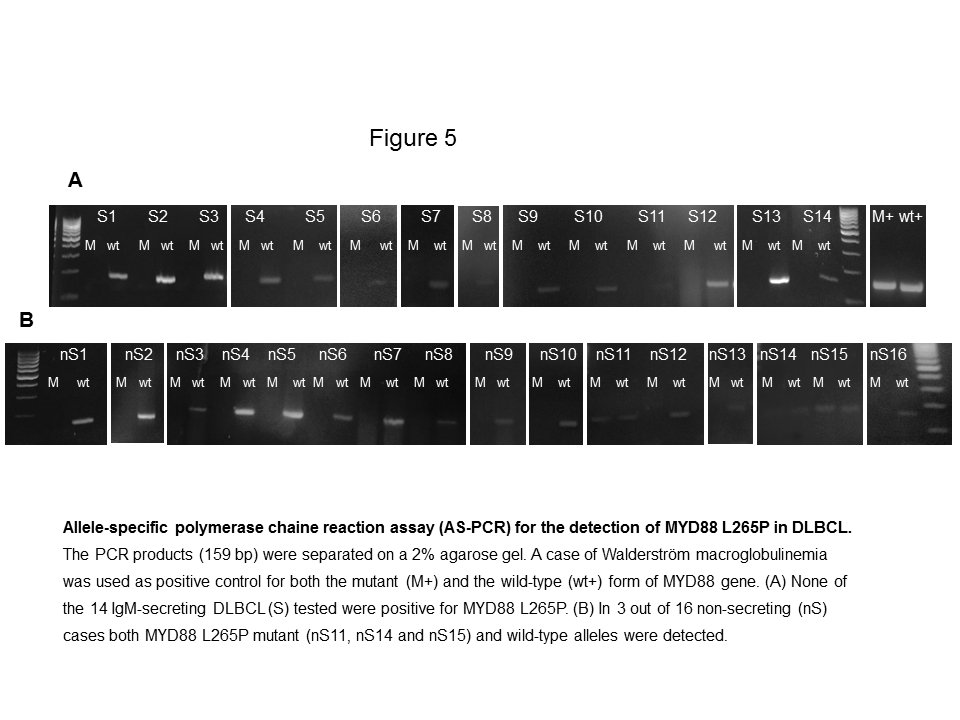


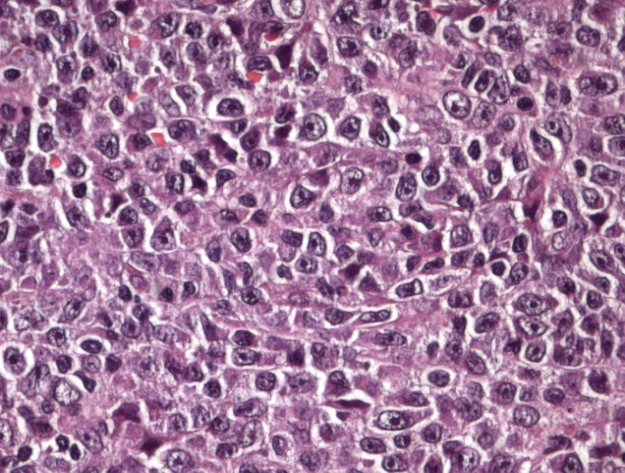

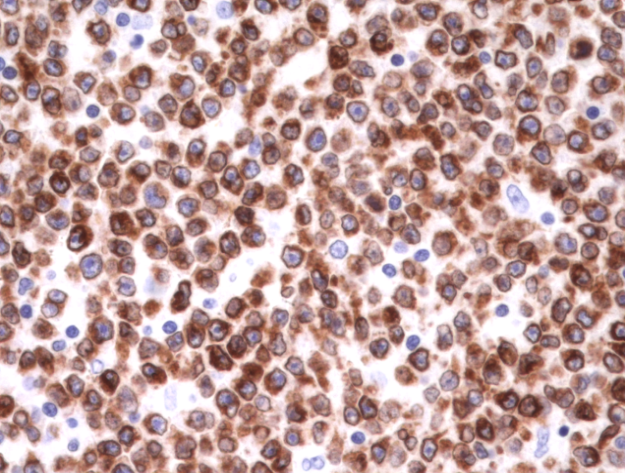

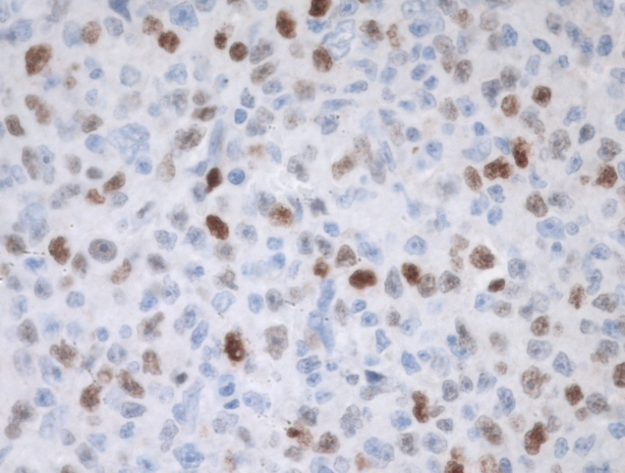

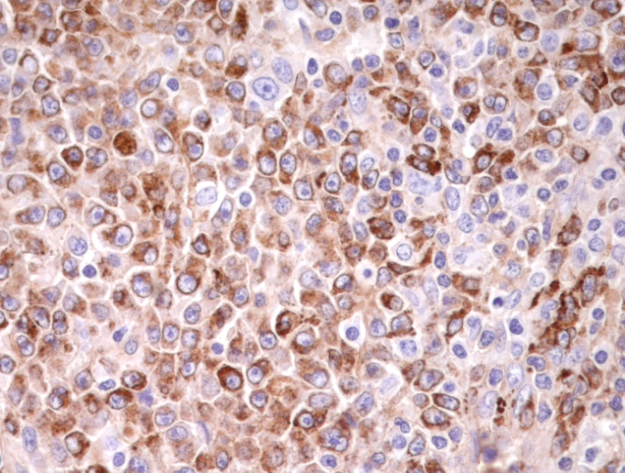


**A**

**B**

**C**

**D**

Legend for figure S1

IgM-secreting DLBCL. Most neoplastic cells show atypical immunoblastic morphology (A), and stain for cytoplasmic IgM (B) and BCL-2 (C): nuclear staining for MYC is present only in a fraction of the neoplastic cells (D) (Immunoperoxidase, x400).

**Figure S2**

**Table S1**: FISH and molecular analyses in DLBCL patients

| **Test** | **Result** | **All cases** | **Ig-M secreting** | **Non-secreting** | **P-value** |
| --- | --- | --- | --- | --- | --- |
| **MYD88**^1^ |  | **N=30** | **N=14** | **N=16** |  |
|  | ***Wild type*** | 27(90%) | 14 (100%) | 13 (81%) |  |
|  | ***Mutated*** | 3(10%) | 0 (0%) | 3(19%) | .244 |
| **MYC**^2^ |  | **N=45** | **N=13** | **N=32** |  |
|  | ***not translocated*** | 42 (93.4%) | 13(100%) | 29 (92.7%) |  |
|  | ***translocated*** | 3 (6.6%) | 0 (0%) | 3(9.3%) | .544 |
| **BCL2**^3^ |  | **N=38** | **N=13** | **N=25** |  |
|  | ***not translocated*** | 30 (79%) | 11 (84.7%) | 19 (76%) |  |
|  | ***translocated*** | 8 (21%) | 2 (15.3%) | 6 (24%) | .689 |
| **BCL6**^4^ |  | **N=38** | **N=12** | **N=26** |  |
|  | ***not translocated*** | 30 (79%) | 11(91.7%) | 19(73%) |  |
|  | ***translocated*** | 8 (21%) | 1 (8.3%) | 7 (27%) | .393 |
| **EBER** |  | **N=36** | **N=15** | **N=21** |  |
|  | ***negative*** | 36 (100%) | 15 (100%) | 21 (100%) |  |
|  | ***positive*** |  |  |  | .567 |

Legend for Table S1

MYD88^1^ : MYD88 gene analysed for L265P mutation

MYC^2^: MYC gene translocation by FISH analysis

BCL2^3^ : BCL2 gene translocation by FISH analysis

BCL6^4^: BCL6 gene translocation by FISH analysis

**Results of BCL2/MYC proteins expression:**

The assessment of BCL2 and MYC protein expression by ICH was carried out in 80 and 37 patients respectively. In this series we could not find a cut-off value of BCL2 and c-MYC that was of prognostic value for survival analyses (data not shown). Since the median percentage of BCL2-positive lymphoma cells was 70%, and the median percentage of c-MYC-positive tumor cells was 40%, the median scores were used as cut-offs to define cases as double-hit (DHT-I). Within the IgM-secreting group seven patients (43%) were scored as double-hit positive for BCL2 and MYC protein expression, nine were scored as negative and one was not evaluated (Table 2).

In the IgM-secreting subset double-positivity for BCL2 and MYC proteins expression was not a significant factor for survival (Figure S6).

**Figure S3**

Progression Free Survival (PFS)


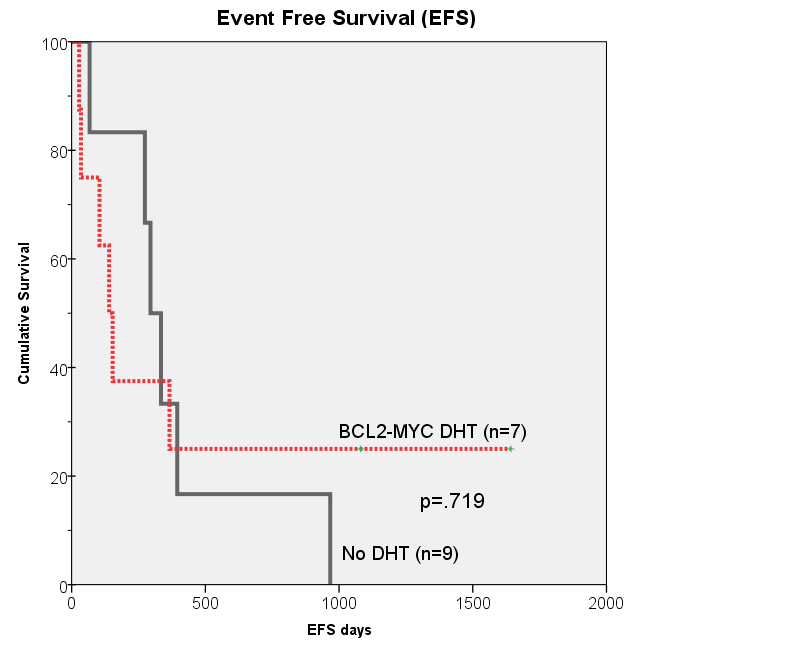


PFS days

Legend for Figure S3

Kaplan-Meier estimates of progression free survival (PFS) in IgM-secreting DLBCL patients positive and negative for BCL2-MYC double-hit score (DHT).
